# Noncoding RNAs in Neurodegenerative Diseases

**DOI:** 10.1155/2013/375852

**Published:** 2013-04-30

**Authors:** Shraddha D. Rege, Thangiah Geetha, Satyanarayana R. Pondugula, Claire A. Zizza, Catherine M. Wernette, Jeganathan Ramesh Babu

**Affiliations:** ^1^Department of Nutrition, Dietetics, and Hospitality Management, Auburn University, Auburn, AL 36849, USA; ^2^Department of Physical Sciences, Auburn University at Montgomery, Montgomery, AL 36117, USA; ^3^Department of Anatomy, Physiology and Pharmacology, Auburn University, Auburn, AL 36849, USA

## Abstract

Noncoding RNAs are widely known for their various essential roles in the development of central nervous system. It involves neurogenesis, neural stem cells generation, maintenance and maturation, neurotransmission, neural network plasticity, formation of synapses, and even brain aging and DNA damage responses. In this review, we will discuss the biogenesis of microRNA, various functions of noncoding RNA's specifically microRNAs (miRNAs) that act as the chief regulators of gene expression, and focus in particular on misregulation of miRNAs which leads to several neurodegenerative diseases as well as its therapeutic outcome. Recent evidences has shown that miRNAs expression levels are changed in patients with neurodegenerative diseases; hence, miRNA can be used as a potential diagnostic biomarker and serve as an effective therapeutic tool in overcoming various neurodegenerative disease processes.

## 1. Introduction

Genetic and environmental factors can contribute to the development of neurodegenerative diseases. Recent studies demonstrate roles for regulatory noncoding RNA molecules (ncRNAs) in normal CNS development and function and in the onset and progression of various neurodegenerative diseases. ncRNAs are functional RNA molecules expressed specifically in the central nervous system that do not encode proteins. They are classified as small ncRNAs comprising fewer than 400 nucleotides and long ncRNAs comprising more than 400 nucleotides. Small ncRNAs include ribosomal RNAs (rRNAs), transfer RNAs (tRNAs), small nucleolar RNAs (snRNAs), microRNAs (miRNAs), short small interference RNAs (siRNAs), and piwi-interacting RNAs. Long noncoding RNAs (lncRNAs) include heterogeneous regulatory molecules such as long intergenic noncoding RNAs (linc RNAs) and natural antisense transcripts (NATs) [1–3]. ncRNAs play critical roles in neuronal processes such as transcription of neuronal genes, brain morphogenesis, neuronal cell specification, and formation of memory [3]. 

## 2. Biogenesis of MicroRNAs

MicroRNAs (miRNAs) are small RNA molecules (21–23 nucleotides) involved in the regulation of gene expression that bind posttranscriptionally to the 3′-untranslated region of target mRNAs and either inhibit translation or degrade the target mRNA [4]. A single-stranded RNA, miRNA, is derived from a 70–100-nucleotide hairpin precursor called pre-miRNA that plays a key role in posttranscriptional regulation of target gene expression. Due to their small size, miRNAs are potentially a valuable tool for therapy of cancer, neurodegenerative, and cardiovascular diseases [5, 6]. The miRNAs are transcribed by either RNA polymerase II or III in the nucleus to yield primary transcripts (pri-miRNAs) of different lengths that are processed by the Drosha/DGCR8 enzyme complex into a ~70 base pair precursor miRNA [7]. The precursor miRNAs are transported into the cytoplasm where cytoplasmic RNase III enzymes, Dicer and Loquacious, process them into ~22-nucleotide miRNA duplexes with guide and passenger strands. The guide strand functions as a mature miRNA and is incorporated into an RNA-induced silencing complex (RISC). This complex contains an Argonaute (Ago) protein as a primary component that binds to the target mRNA and degrades the passenger strand. Mature miRNA guides RISC to recognize target sequences located in the 3′UTR of mRNAs, which leads to the inhibition of translation or degradation of mRNA [6, 7].

## 3. The Role of miRNAs in Neurodegenerative Diseases

Neurodegeneration refers to the death of neurons during the normal course of brain development, and many studies demonstrate the involvement of miRNAs. The miRNAs are key regulators of CNS development involving brain plasticity, neuronal cell maturation, neuronal cell differentiation, and synaptogenesis [8, 9]. Dysregulation of miRNAs leads to the development of neurodegenerative diseases such as Alzheimer's disease, Parkinson's disease, and Huntington's disease [9]. 

### 3.1. miRNAs in Alzheimer's Disease

Alzheimer's disease (AD) is the most common form of dementia, among elderly adults, and causes memory impairment as well as problems with thinking, decision-making, and behavior. AD is characterized by dysregulated processing of amyloid precursor protein (APP) and the accumulation of amyloid beta (A*β*) peptide in brain hippocampus [3]. *β*-secretase, also known as *β*-site APP-cleaving enzyme 1 (BACE 1), cleaves APP to generate A*β* peptides [10]. Several studies show that increased expression of APP, an integral membrane protein, is associated with AD. MicroRNAs play an important role in APP regulation [11]. Vilardo et al. [12] showed that miR-101 negatively regulates the expression of APP and A*β* accumulation in cultured hippocampal neurons, thus suggesting an essential role for miRNA in the development and progression of AD [12]. Postmortem studies of human brain hippocampus showed increased levels of BACE1, upregulation of miR-9, miR-125B, and miR-125b, MIr-146a, and downregulation of miR-15a, miR-107, and miR-29a/b-1, which suggests a link between miRNAs and protease in the progression of the disease [3, 9]. Decrease in the expression of miR-107 was observed in patients who exhibit early stages of AD. Affymetrix Exon Array microarrays demonstrated a sharp increase in BACE1 mRNA expression and a decrease in miR-107 expression [13]. In addition, the miR-29a/b-1 cluster is down-regulated in AD patient's brain while upregulation of BACE1 is observed, which suggests a role for the miR-29a/b-1 cluster in negative regulation of BACE1 expression that leads to the accumulation of A*β* peptide [14]. Certain patterns of miRNA expression in the grey matter of cortex also lead to AD pathogenesis. MiR-211 is downregulated in Alzheimer's disease brain, whereas there is an upregulation of miR-424 [15]. 

A murine model of AD showed that there was no correlation between BACE1 mRNA and levels of proteins in the hippocampus of APPSwe/PS1 mice [16]. A study by Boissonneault et al. [16] supports the concept that there is an association between dysfunctional miRNA regulation of BACE1 expression and AD by further analysis of miR-298 and miR-328 in the hippocampus of APPSwe/PS1 mice [16]. In addition, a novel noncoding RNA 17A expressed in human brain is upregulated in cerebral tissues of AD patients and leads to increased secretion of A*β* peptides, GPR51 alternative splicing, and ultimately, impaired GABA B function [17]. A similar study showed that Neuroblastoma Differentiation Marker 29 (NDM29), a RNA polymerase III-dependent noncoding RNA, enhances the synthesis of amyloid precursor protein, eventually resulting in an increase of A*β* secretion that promotes the formation of A*β* peptides as they may occur in Alzheimer's disease [18]. NF-*κ*B signaling regulates miR-146a, which is upregulated in AD brains, and downregulation of CFH protein is observed in AD brains, which suggests that miR-146a targets CFH protein, a brain inflammatory response repressor [19].

### 3.2. miRNAs in Parkinson's Disease

The death of dopamine generating cells in the brain substantia nigra and accumulation of alpha-synuclein leads to the development of Parkinson's disease, which is characterized by motor abnormalities and sensory, mood, and cognitive changes [20]. Specific miRNAs control *α* synuclein expression. A study suggests that *α*-synuclein is regulated posttranscriptionally by miR-7 and miR-153, which bind directly to the 3′-untranslated region of *α* synuclein and suppress its expression. In addition, downregulation of *α* synuclein due to miR-7 and miR-153 protects cells from oxidative stress. In primary neurons, increased translation of a luciferase construct that bears *α* synuclein 3′-untranslated region was reportedly caused by inhibition of micro-RNAs miR-7 and miR-153 [21]. Elevated levels of fibroblast growth factor 20 (FGF20) genes also lead to overexpression of *α* synuclein and eventually to the death of dopaminergic neurons [13]. Furthermore, this demonstrates that binding of miR-433 to a single nucleotide polymorphism in the promoter region of FGF20 disrupts the target site for miR-433, which eventually results in overexpression of *α* synuclein [13]. Downregulation of miR-7 in the 1-methyl-4-phenyl-1,2,3,6-tetrahydropyridine-(MPTP-) induced neurotoxin PD mouse model suggests that it may protect cells against oxidative stress [20]. The most vital gene in Parkinson's disease, LRRK2 that functions in the dopamine generating cells negatively regulates let-7 and mir-184, which causes overexpression of their targets E2F1 and DP eventually, leads to defects in cell division and cell death [22]. Expression of miR-133b is detected in the midbrain dopaminergic neurons but not detected in Parkinson's disease midbrain tissue. A negative feedback loop with the transcription factor PITX3, found in midbrain dopamine generating neurons, is formed by the downregulation of mir-133b, which is a dopaminergic neuron-specific miRNA [4]. Downregulation of mir-133b may play a neuroprotective role. Furthermore, a recent study suggests that overexpression of *α* synuclein by miR-433 leads to increased risk of AD [13]. Also, activation of two neuroprotective genes PTEN-induced putative kinase (PINK1) and Parkinson disease 7 (PARK7) normally involved in protecting cells from oxidative stress leads to development of Parkinson's disease through PTEN signaling [23]. 

### 3.3. miRNAs in Huntington's Disease

Huntington's disease is an autosomal dominant neurodegenerative disease characterized by cognitive decline and impaired movement. It is caused by trinucleotide expansion in the gene that encodes a polyglutamine stretch near the N-terminus of huntingtin [24]. Multiple studies of miRNAs in mouse and human brain have shown miR-22, miR-29c, and miR-128 are down-regulated in mice and miR-9/9*, miR-29b, and miR-124a are down-regulated in humans [25, 26]. Marti et al. showed that miR-221 and miR-222 are both down-regulated in HD. Also, downregulation of miRNAs let-7a, let-7c, let-7d, and let 7e was observed in HD-FC (frontal cortex) and HD-ST (striatum) whereas there was upregulation of miR-30b, miR-30c, and miR-30e in HD-FC and HD-ST [27]. The miR-30a, that is, upregulated in HD-FC and HD-ST has been shown to target BDNF, which is a REST-modulated neuronal gene. Loss of BDNF leads to loss of medium spiny neurons [28, 29] REST/CoREST regulates many miRNAs. REST repressor complex regulates miR-9 and miR-9* that target REST/CoREST expression and are considered to be a part of a double-negative feedback loop [3]. Expression of miR-9/9*, miR-124a, and miR-132 is regulated by the REST repressor complex including mSin3, REST corepressor 1 (COREST), and MeCP2. REST silencing complex and miR-9/9* give rise to a negative feedback loop during the development of Huntington's disease due to the effects of miR-9 and miR-9* on REST and COREST. Several miRNAs such as miR-9/9*, miR-129a, miR-132, miR-29b, miR-29a, miR-330, miR-17, miR-196, miR-222, miR-485, and miR-486 are affected in HD due to the disrupted miRNA transcriptome [27, 30]. REST targets miR-9 and miR-124 [27]. It was shown that miR-184 expressed in neurons is upregulated due to depolarization [31]. Moreover, reports suggest that MiR-34b is elevated in plasma of Huntington's disease patients even before the onset of symptoms, which implies that miR-34b may be useful as a potential diagnostic biomarker for HD [32, 33]. 

## 4. Applications/Therapeutic Role

Clearly, there is evidence for miRNAs as potential biomarkers to aid in the diagnosis of neurodegenerative diseases [34]. For instance, RNA interference (RNAi) knocks down the translation of certain disease-associated molecules due to the presence of short hairpin RNAs, synthetic double-stranded RNAs, and the precursors of artificial miRNAs that are expressed by viral vectors [35]. Similarly, several RNA-based drugs have been formulated to target various diseases. These drugs are synthetic RNA molecules that mimic a mechanism of action similar to that of endogenous ncRNAs—siRNAs or miRNAs [35]. Thus, RNA interference technology has emerged as a potential therapeutic tool for several neurodegenerative diseases. Wang et al. [36] suggest the following two different potential roles of miR-146: (1) as a useful biomarker in the early diagnosis of AD since it can be detected in human blood monocytes, which aids in easy collection, and (2) as a therapeutic tool. They also suggest that anti-miR-146a can be used with therapeutic potential to suppress the up-regulatory effects of miR-146 [36]. Another study conducted to examine the pattern of A*β* driven neurogenesis in a transgenic mouse model of AD-Tg-19959 showed that the use of LNA-modified siRNAs led to a marked decrease in the levels of insoluble A*β* peptides and altered the pattern of aggregation of soluble A*β* in the Tg-19959 mouse brain by targeting BACE1 and BACE-AS. This was considered the most useful potential tool for early diagnosis of AD [37]. Cogswell et al. [38] identified two diagnostic biomarkers: dysregulation of miRNA in AD patients' brains and changes in miRNA specific to AD in cerebrospinal fluid [38]. Several miRNAs such as miR-133b, miR-7, miR-153, let-7, miR184, and miR-433 may be involved in the pathophysiology of PD and offer a novel approach for therapeutic targets. In addition, the miRNA pathway is known to play a crucial role in translational regulation in the pathophysiology of PD and hence is considered one of the most important translation regulation pathways [5]. 

Harper et al. (2005) showed notable signs of improvement in the behavioral abnormalities and neuropathology among HD patients with the use of an adenoassociated virus serotype 1 (AAV1) vector that targets the mutant human htt in the brain of mouse giving rise to U6 promoter-driven shRNA effector system. This is also considered as a potential therapeutic approach in HD [39]. In addition, noncoding small RNA-based technology plays a pivotal role in the treatment of Huntington's disease [24].

Antisense oligonucleotides (Aos) help to prevent mRNAs bearing mutations by redirecting pre-mRNA splicing. Aos thereby emerge as a potential therapeutic tool and act to regulate alternative splicing [40]. Overall, data suggest that various noncoding RNAs could serve as useful diagnostic biomarkers and therapeutic targets for neurodegenerative diseases.

## 5. Future Projections

ncRNAs, especially miRNAs, are known for their roles in normal development and function of the central nervous system. They are involved in targeted gene expression and act as key regulators in several neuroprotective mechanisms. It is a well-established fact that dysregulation of miRNAs eventually leads to the development of various neurodegenerative diseases. Alterations in the patterns of miRNA expression will probably serve as diagnostic biomarkers of brain function, and the initiation and progression of neurological disorders and neurodegenerative diseases [41]. Further research in miRNA expression patterns and profiling will result in the discovery of many more novel biomarkers. In-depth study to determine the functions of long noncoding RNAs in RNA-mediated gene regulation is likely to remain an area of intense research interest [9]. 

## References

[1] Qureshi IA, Mattick JS, Mehler MF (2010). Long non-coding RNAs in nervous system function and disease. *Brain Research*.

[2] Mattick JS (2011). The central role of RNA in human development and cognition. *FEBS Letters*.

[3] Salta E, Strooper BD (2012). Non-coding RNAs with essential roles in neurodegenerative disorders. *The Lancet Neurology*.

[4] Kim J, Inoue K, Ishii J (2007). A microRNA feedback circuit in midbrain dopamine neurons. *Science*.

[5] Harraz MD, Dawson TM, Dawson VL (2011). MicroRNAs in Parkinson's disease. *Journal of Chemical Neuroanatomy*.

[6] Liu NK, Xu XM (2011). MicroRNA in central nervous system trauma and degenerative disorders. *Physiological Genomics*.

[7] Bartel DP (2009). MicroRNAs: target recognition and regulatory functions. *Cell*.

[8] Nelson PT, Wang WX, Rajeev BW (2008). MicroRNAs (miRNAs) in neurodegenerative diseases. *Brain Pathology*.

[9] Bian S, Sun T (2011). Functions of noncoding RNAs in neural development and neurological diseases. *Molecular Neurobiology*.

[10] Provost P (2010). MicroRNAs as a molecular basis for mental retardation, Alzheimer’s and prion diseases. *Brain Research*.

[11] Du L, Pertsemlidis A (2011). Cancer and neurodegenerative disorders: pathogenic convergence through microRNA regulation. *Journal of Molecular Cell Biology*.

[12] Vilardo E, Barbato C, Ciotti M, Cogoni C, Ruberti F (2010). MicroRNA-101 regulates amyloid precursor protein expression in hippocampal neurons. *The Journal of Biological Chemistry*.

[13] Wang G, van der Walt JM, Mayhew G (2008). Variation in the miRNA-433 binding site of FGF20 confers risk for parkinson disease by overexpression of *α*-synuclein. *American Journal of Human Genetics*.

[14] Hebert SS, Horre K, Nicolai L (2008). Loss of microRNA cluster miR-29a/b-1 in sporadic Alzheimer's disease correlates with increased BACE1/beta-secretase expression. *Proceedings of the National Academy of the Sciences of the United States of America*.

[15] Wang WX, Huang Q, Hu Y, Stromberg AJ, Nelson PT (2011). Patterns of microRNA expression in normal and early Alzheimer’s disease human temporal cortex: white matter versus gray matter. *Acta Neuropathologica*.

[16] Boissonneault V, Plante I, Rivest S, Provost P (2009). MicroRNA-298 and microRNA-328 regulate expression of mouse *β*-amyloid precursor protein-converting enzyme 1. *The Journal of Biological Chemistry*.

[17] Massone S, Vassallo I, Fiorino G (2011). 17A, a novel non-coding RNA, regulates GABA B alternative splicing and signaling in response to inflammatory stimuli and in Alzheimer disease. *Neurobiology of Disease*.

[18] Massone S, Ciarlo E, Vella S (2012). NDM29, a RNA polymerase III-dependent non-coding RNA, promotes amyloidogenic processing of APP and amyloid *β* secretion. *Biochimica et Biophysica Acta*.

[19] Lukiw WJ, Zhao Y, Jian GC (2008). An NF-*κ*B-sensitive micro RNA-146a-mediated inflammatory circuit in alzheimer disease and in stressed human brain cells. *The Journal of Biological Chemistry*.

[20] Junn E, Lee KW, Byeong SJ, Chan TW, Im JY, Mouradian MM (2009). Repression of *α*-synuclein expression and toxicity by microRNA-7. *Proceedings of the National Academy of Sciences of the United States of America*.

[21] Doxakis E (2010). Post-transcriptional regulation of *α*-synuclein expression by mir-7 and mir-153. *The Journal of Biological Chemistry*.

[22] Gehrke S, Imai Y, Sokol N, Lu B (2010). Pathogenic LRRK2 negatively regulates microRNA-mediated translational repression. *Nature*.

[23] Morris LGT, Veeriah S, Chan TA (2010). Genetic determinants at the interface of cancer and neurodegenerative disease. *Oncogene*.

[24] Zhang Y, Friedlander RM (2011). Using non-coding small RNAs to develop therapies for Huntington's disease. *Gene Therapy*.

[25] Johnson R, Buckley NJ (2009). Gene dysregulation in Huntington’s disease: REST, microRNAs and beyond. *Neuromolecular Medicine*.

[26] Lee ST, Chu K, Im WS (2011). Altered microRNA regulation in Huntington’s disease models. *Experimental Neurology*.

[27] Marti E, Pantano L, Banez-Coronel M (2010). A myriad of miRNA variants in control and Huntington’s disease brain regions detected by massively parallel sequencing. *Nucleic Acids Research*.

[28] Zuccato C, Ciammola A, Rigamonti D (2001). Loss of huntingtin-mediated BDNF gene transcription in Huntington’s disease. *Science*.

[29] Mellios N, Huang HS, Grigorenko A, Rogaev E, Akbarian S (2008). A set of differentially expressed miRNAs, including miR-30a-5p, act as post-transcriptional inhibitors of BDNF in prefrontal cortex. *Human Molecular Genetics*.

[30] Johnson R, Zuccato C, Belyaev ND, Guest DJ, Cattaneo E, Buckley NJ (2008). A microRNA-based gene dysregulation pathway in Huntington’s disease. *Neurobiology of Disease*.

[31] Nomura T, Kimura M, Horii T (2008). MeCP2-dependent repression of an imprinted miR-184 released by depolarization. *Human Molecular Genetics*.

[32] Conaco C, Otto S, Han JJ, Mandel G (2006). Reciprocal actions of REST and a microRNA promote neuronal identity. *Proceedings of the National Academy of Sciences of the United States of America*.

[33] Packer AN, Xing Y, Harper SQ, Jones L, Davidson BL (2008). The bifunctional microRNA miR-9/miR-9* regulates REST and CoREST and is downregulated in Huntington’s disease. *Journal of Neuroscience*.

[34] Mack GS (2007). MicroRNA gets down to business. *Nature Biotechnology*.

[35] Cooper TA, Wan L, Dreyfuss G (2009). RNA and disease. *Cell*.

[36] Wang LL, Huang Y, Wang G, Chen SD (2012). The potential role of microRNA-146 in Alzheimer's disease: biomarker or therapeutic?. *Medical Hypotheses*.

[37] Modarressi F, Faghihi MA, Patel NS, Sahagan BG, Wahlestedt C, Lopez-Toledano MA (2011). Knockdown of BACE1-AS Nonprotein-Coding Transcript modulates beta- amyloid related hippocampal neurogenesis. *International Journal of Alzheimer’s Disease*.

[38] Cogswell JP, Ward J, Taylor IA (2008). Identification of miRNA changes in Alzheimer’s disease brain and CSF yields putative biomarkers and insights into disease pathways. *Journal of Alzheimer’s Disease*.

[39] Harper SQ, Staber PD, He X (2005). RNA interference improves motor and neuropathological abnormalities in a Huntington’s disease mouse model. *Proceedings of the National Academy of Sciences of the United States of America*.

[40] Sazani P, Kole R (2003). Modulation of alternative splicing by antisense oligonucleotides. *Progress in Molecular and Subcellular Biology*.

[41] Feng W, Feng Y (2011). MicroRNAs in neural cell development and brain diseases. *Science China Life Sciences*.

